# Combination of Systemic and Lock-Therapies with Micafungin Eradicate Catheter-Based Biofilms and Infections Caused by *Candida albicans* and *Candida parapsilosis* in Neutropenic Rabbit Models

**DOI:** 10.3390/jof10040293

**Published:** 2024-04-17

**Authors:** Ruta Petraitiene, Vidmantas Petraitis, Myo H. Zaw, Kaiser Hussain, Rodolfo J. Ricart Arbona, Emanuel Roilides, Thomas J. Walsh

**Affiliations:** 1 Transplantation-Oncology Infectious Diseases Program, Division of Infectious Diseases, Department of Medicine, Weill Cornell Medicine of Cornell University, 1300 York Ave., New York, NY 10065, USA; vip2007@med.cornell.edu (V.P.); myohtet111@gmail.com (M.H.Z.); khussain@houstonmethodist.org (K.H.); 2 Sutter Health Memorial Medical Center, 1700 Coffee Rd., Modesto, CA 95355, USA; 3 Department of Radiology, Houston Methodist Hospital, Houston Radiology Associated, 6565 Fannin St. #268, Houston, TX 77030, USA; 4 Center for Comparative Medicine and Pathology, Memorial Sloan Kettering Cancer Center and Weill Cornell Medicine, 1275 York Ave., New York, NY 10021, USA; 5 Department of Genetic Medicine, Weill Cornell Medicine of Cornell University, 1300 York Ave., New York, NY 10065, USA; 6 Hippokration Hospital, School of Medicine, Aristotle University, Konstantinoupoleos 49, GR-54642 Thessaloniki, Greece; roilides@auth.gr; 7 Center for Innovative Therapeutics and Diagnostics, Richmond, VA 23220, USA

**Keywords:** micafungin, *Candida albicans*, *Candida parapsilosis*, biofilm, catheter, lock therapy, (1→3)-β-D-glucan, rabbit, neutropenic

## Abstract

Vascular catheter-related infections, primarily caused by *Candida albicans* and *Candida parapsilosis*, pose significant challenges due to the formation of biofilms on catheters, leading to refractory disease and considerable morbidity. We studied the efficacy of micafungin in systemic and lock therapies to eliminate catheter-based biofilms and deep tissue infections in experimental central venous catheter (CVC)-related candidemia in neutropenic rabbits. Silastic CVCs in rabbits were inoculated with 1 × 10^3^ CFU/mL of *C. albicans* or *C. parapsilosis*, establishing catheter-based biofilm, and subjected to various treatments. Neutropenic rabbits treated with a combination of lock therapy and systemic micafungin demonstrated the most significant reduction in fungal burden, from 5.0 × 10^4^ to 1.8 × 10^2^ CFU/mL of *C. albicans* and from 5.9 × 10^4^ to 2.7 × 10^2^ CFU/mL of *C. parapsilosis* (*p* ≤ 0.001), in the CVC after 24 h, with full clearance of blood cultures after 72 h from treatment initiation. The combination of lock and systemic micafungin therapy achieved eradication of *C. albicans* from all studied tissues (0.0 ± 0.0 log CFU/g) vs. untreated controls (liver 7.5 ± 0.22, spleen 8.3 ± 0.25, kidney 8.6 ± 0.07, cerebrum 6.3 ± 0.31, vena cava 6.6 ± 0.29, and CVC wash 2.3 ± 0.68 log CFU/g) (*p* ≤ 0.001). Rabbits treated with a combination of lock and systemic micafungin therapy demonstrated a ≥2 log reduction in *C. parapsilosis* in all treated tissues (*p* ≤ 0.05) except kidney. Serum (1→3)-β-D-glucan levels demonstrated significant decreases in response to treatment. The study demonstrates that combining systemic and lock therapies with micafungin effectively eradicates catheter-based biofilms and infections caused by *C. albicans* or *C. parapsilosis*, particularly in persistently neutropenic conditions, offering promising implications for managing vascular catheter-related candidemia and providing clinical benefits in cases where catheter removal is not feasible.

## 1. Introduction

Central line-associated bloodstream infection (CLABSI) and catheter-related blood stream infection (CRBSI) caused by *Candida* spp. constitutes a common source of nosocomial candidemia, necessitating prompt removal of the vascular catheter to control the infection, prevent hemodynamic deterioration, and improve survival [1,2,3,4,5,6]. Unfortunately, central lines, particularly in patients with thrombocytopenia, coagulopathies, or limited venous access, often cannot be removed. A critical need exists for a comprehensive strategy for management of candidemia and CRBSI in these vulnerable patients. Such a strategy would enable the retention of central venous catheters, including central Silastic venous catheters (Hickman^®^, Broviac^®^) and portacaths. As the removal and replacement of these vascular catheters may be fraught with serious morbidity and potential mortality, developing strategies for treatment of *Candida*-related central venous catheters becomes paramount in advancing the care of patients with CRBSI caused by *Candida* spp.

*Candida albicans* and *Candida parapsilosis* are significant causes of CRBSIs in pediatric and adult patients [7,8,9,10,11]. These pathogens form biofilms that adhere to vascular catheters, resulting in clinically refractory candidemia and significant morbidity [12]. *Candida* biofilms are structured communities attached to surfaces and enclosed in an exopolymeric matrix. Biofilms of *C. albicans* consists of a basal layer of blastoconidia with a dense overlying matrix composed of hyphae, exopolysaccharides, organism- and host-derived proteins, lipids, and DNA [13,14,15]. These biofilm cells were metabolically active and found to be resistant to fluconazole [12].

Echinocandins exert potent in vitro fungicidal activity against *Candida* spp. and *Candida* biofilms [16,17,18,19]. The efficacy of caspofungin, anidulafungin, and micafungin against *Candida* biofilms in vascular catheters was evaluated in murine model studies [20,21,22,23,24,25]. Several studies investigated the efficacy of caspofungin or anidulafungin in rabbit models with vascular catheters [26,27,28]. In order to develop a preclinical foundation for echinocandins in the medical management of *Candida* CRBSIs in patients whose vascular catheters cannot be removed, we studied the efficacy of micafungin for treatment of experimental central venous catheter (CVC) infection in a rabbit model of disseminated candidiasis. We studied micafungin as a model for all licensed echinocandins in these experiments. Although we also have previously studied caspofungin and anidulafungin in our rabbit models, we have studied micafungin more extensively in vitro and in vivo [29,30,31,32] and in clinical research [33,34]. Moreover, as micafungin is the echinocandin used in our institution for treatment of patients with invasive candidiasis, we investigated this echinocandin with the possibility of translating these findings to clinical protocols for patient care.

We hypothesized that micafungin, as a representative echinocandin, when administered as lock therapy would eradicate CVC biofilm-based *Candida* infection, that systemic therapy would reduce residual deep tissue burden, and that only the combination of lock therapy and systemic administration of micafungin would achieve the successful eradication of CVC infection and a significant reduction in residual deep tissue burden.

## 2. Materials and Methods

### 2.1. Animals

Female New Zealand white rabbits (weight 2.6 to 3.3 kg; Covance Research Products, Inc., Denver, PA, USA) were used in the studies. According to the Guide for the Care and Use of Laboratory Animals [35], the rabbits were housed individually in the Research Animal Resource Center (RARC) laboratory animal facilities at Weill Cornell Medicine (WCM), and water and standard rabbit feed were provided *ad libitum*. WCM is accredited by the Association for the Assessment and Accreditation of Laboratory Animal Care International (AAALAC). The Institutional Animal Care and Use Committee (IACUC) at WCM approved the study.

The indwelling subcutaneous Silastic central venous catheters were placed at least 72 h before the studies in all animals while they were under general anesthesia [36]. The Silastic catheter permitted nontraumatic venous access for the administration of *Candida* inoculum and parental agents, and for repeated blood sampling. The rabbits were euthanized according to the IACUC-approved pre-specified humane endpoints by intravenous administration of pentobarbital (65 mg of pentobarbital sodium/kg of body weight; SomnaSol™ Euthanasia-III Solution C3N; Henry Schein Animal Health, Dublin, OH, USA) or at the end of the study.

### 2.2. Study Drugs

Micafungin (Astellas Pharma US, Inc., Deerfield, IL, USA) was provided as a sterile powder and was reconstituted and diluted further in sterile 0.9% normal saline to achieve the required concentrations of 1 mg/mL or 10 mg/mL.

The dosage of systemically administered micafungin was based upon prior laboratory investigations and preliminary data for these experiments [29,30,31,32]. The concentrations for lock therapy were derived from previous in vitro studies conducted in our laboratory [17,18].

The minimal inhibitory concentrations (MICs) of micafungin were determined by standard methods of the Clinical and Laboratory Standards Institute (CLSI) using broth microdilution [37] and were defined as the lowest concentration that caused an optically clear well. The MICs of micafungin against *C. albicans* and *C. parapsilosis* were 0.125 μg/mL and 0.125 μg/mL, respectively.

### 2.3. Organism, Inoculum, and Inoculation

#### 2.3.1. *Candida albicans*

A well-characterized clinical *C. albicans* isolate NIH-8621 (ATCC MYA-1237) from a neutropenic patient with autopsy-proven disseminated candidiasis was used for all studies. The inoculum was prepared as described previously in [29,31]. An inoculum of 1 × 10^3^ blastoconidia of *C. albicans* was inoculated to the Silastic catheter in a volume of 200 µL (volume of the inoculum could be “locked” in the internal lumen of the catheter to establish the development of the biofilm). The inoculum size was confirmed by plating serial dilutions onto SGA plates.

#### 2.3.2. *Candida parapsilosis*

Clinical *C. parapsilosis* isolate NIH-16 obtained from a neutropenic patient with autopsy-proven disseminated candidiasis was used in the studies. The inoculum was prepared using the same method as for *C. albicans* isolate. An inoculum of 1 × 10^6^ blastoconidia of *C. parapsilosis* was inoculated to the Silastic catheter in a volume of 200 µL (the volume of the inoculum could be “locked” in the internal lumen of the catheter to establish the development of the biofilm).

### 2.4. Immunosuppression of Rabbits and Maintenance of Neutropenia

Cytarabine (Ara-C; Cytosar-U, Pharmacia, Kalamazoo, MI, USA) was administered to rabbits intravenously for induction and maintenance of neutropenia. Cytarabine sterile solution for injection at 50 mg/mL was used in the studies. Profound and persistent neutropenia (<100/μL) was achieved by an initial course of 440 mg/m^2^ of Ara-C on days 1 through 5 before inoculation of the rabbits with *Candida*. Ara-C was administered at 440 mg/m^2^ at 2-day intervals to maintain profound and persistent neutropenia during the study.

Antibiotics were administered from day 4 of immunosuppression for the prevention of opportunistic bacterial infections during neutropenia. Ceftazidime (75 mg/kg intravenous twice daily; Glaxo Pharmaceuticals, Division of Glaxo Inc., Research Triangle Park, NC, USA), gentamicin (5 mg/kg intravenous every other day; Elkins-Sinn, Inc., Cherry Hill, NJ, USA), and vancomycin (15 mg/kg intravenous daily; Abbott Laboratories, North Chicago, IL, USA) were injected. To prevent antibiotic-associated diarrhea due to *Clostridium spiroforme*, all rabbits received 50 mg of vancomycin per liter of drinking water.

### 2.5. Antifungal Therapy

#### 2.5.1. *Candida albicans*

The study was conducted in two experiments with all study cohorts. The treatment groups consisted of lock therapy with micafungin at 1 mg/mL (Lock1), 10 mg/mL (Lock10), and systemic therapy with intravenous administration of micafungin at 1 mg/kg (MFG1) or untreated control rabbits. For lock therapy, 250 µL of micafungin solution was injected into the lumen of each catheter and allowed to remain for 8 h per day for 7 days, following removal of antifungal solution with blood in a volume of 1 mL. There were six study groups: (1) untreated control rabbits (*n* = 6), (2) Lock1 (*n* = 5), (3) a combination of systemic and lock therapy Lock1+MFG1 (*n* = 5), (4) Lock10 (*n* = 5), (5) a combination of systemic and lock therapy Lock10+MFG1 (*n* = 5), and (6) MFG1 (*n* = 5) every 24 h. Therapy was initiated 24 h post-inoculation and continued for 7 days, and the rabbits were euthanized 24 h after the last dose on day 8 post-inoculation.

#### 2.5.2. *Candida parapsilosis*

The study was conducted in three experiments with all study cohorts. The treatment groups consisted of lock therapy with micafungin at 10 mg/mL (Lock10), systemic therapy with intravenous administration of micafungin at 1 mg/kg (MFG1), and untreated control rabbits. For lock therapy, 250 µL of micafungin solution were injected into the lumen of each catheter and allowed to remain for 8 h per day for 7 days, following removal of antifungal solution with blood in a volume of 1 mL. There were four study groups: (1) untreated control rabbits (*n* = 6), (2) Lock10 (*n* = 6), (3) Lock10+MFG1 -a combination of systemic and lock therapy (*n* = 6), and (4) MFG1 (*n* = 6) every 24 h. Therapy was initiated 24 h post-inoculation and continued for 7 days, and the rabbits were euthanized 24 h after the last dose on day 8 post-inoculation.

### 2.6. Outcome Variables/Rabbit Model of Catheter-Related Disseminated Candidemia

Assessment of antifungal therapy was determined by quantitative clearance of *C. albicans* or *C. parapsilosis* from blood cultures, the central venous catheter, tissues, and serial serum (1→3)-β-D-glucan levels.

#### 2.6.1. Quantitation of *C. albicans* or *C. parapsilosis* in the Blood

The antifungal efficacy in the model of *Candida* catheter-related disseminated candidemia was determined by the quantitative clearance of *C. albicans* from the blood. Blood samples were collected from the venous catheter every day from each rabbit starting 24 h after inoculation before administering the antifungal therapy. Quantitative cultures, measured as colony-forming units (CFU), were performed every day by collecting 1 mL of blood from the venous catheter and plating 1 mL or adequate dilutions of blood onto SGA plates. Log CFU/mL of blood cultures were calculated.

#### 2.6.2. Quantitation of *C. albicans* or *C. parapsilosis* in the Tissues

Antifungal activity in the model of catheter-related disseminated candidiasis was determined by quantitative clearance of *C. albicans* and *C. parapsilosis* from tissues. Representative sections of liver, spleen, kidney, cerebrum, and anterior vena cava were weighed, and each tissue sample was then homogenized (Seward Stomacher^®^ 80; Fisher Scientific, Hampton, NH, USA) in sterile reinforced polyethylene bags (Nasco Whirl-Pak^®^, Fisher Scientific) with sterile 0.9% saline for 30 s.

Each tissue homogenate or vitreous humor specimen was serially diluted 10^−1^ to 10^−4^ in sterile 0.9% normal saline. The aliquots of 100 µL of undiluted tissue homogenate or vitreous humor and of each dilution were separately plated onto Emmon’s modified SGA containing chloramphenicol and gentamicin. Culture plates were incubated at 37 °C for 24 h, after which CFU were counted and the number of CFU/g of tissue was calculated for each organ. Potential carryover of the drug was minimized by serial dilution and by streaking a small-volume (100 µL) aliquot onto a large volume of agar (1 full agar plate/100 µL aliquot). The limit of detection was ≥10 CFU/g or ≥10 CFU/mL, respectively. The culture-negative plates were counted as 0 CFU/g or 0 CFU/mL. Data were graphed as the mean of log_10_ (CFU/g or CFU/mL) ± standard error of the mean (SEM).

#### 2.6.3. (1→3)-β-D-Glucan Levels in Serum

Antifungal efficacy also was evaluated by (1→3)-β-D-glucan levels in serum samples. Blood samples were collected every day from each rabbit, and (1→3)-β-D-glucan levels were determined according to the manufacturer’s specifications, as described previously [31].

### 2.7. Statistical Analysis

The following outcome variables among all experimental groups were compared: quantitative cultures of venous catheter blood (log CFU/mL), quantitative cultures of tissues (log CFU/g), and (1→3)-β-D-glucan level (pg/mL). Comparisons between groups were performed using the Kruskal–Wallis test (nonparametric ANOVA) or Mann–Whitney *U* test, as appropriate. A 2-tailed *p*-value ≤ 0.05 was considered to be statistically significant. Values are expressed as mean and standard error of the means (SEMs).

## 3. Results

### 3.1. Disseminated Candidiasis Caused by C. albicans in a Neutropenic Rabbit Model

#### 3.1.1. Fungal Burden in Central Venous Catheter Blood

**Figure 1:**
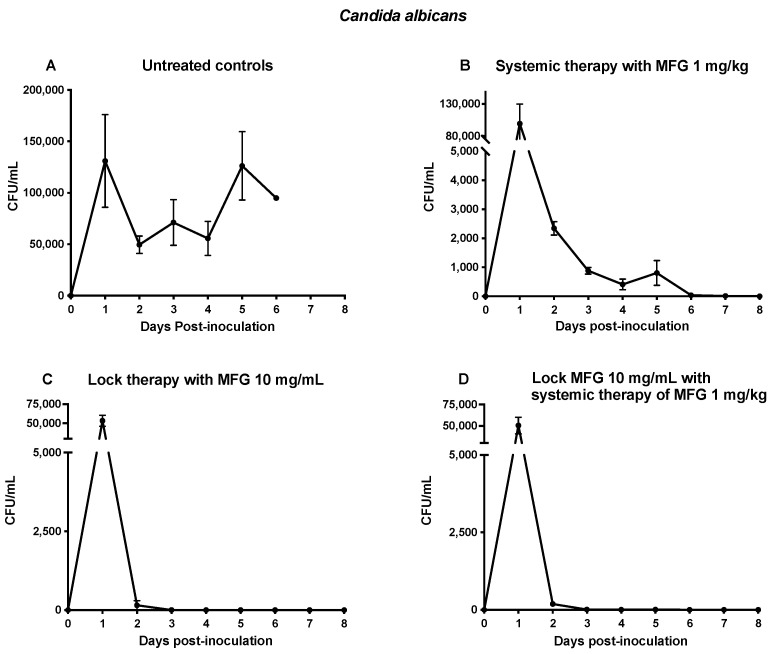
Responses of *C. albicans* fungal burden in disseminated candidiasis in persistently neutropenic rabbits to lock therapy and systemic antifungal therapy measured by fungal burden in blood cultures from untreated control rabbits (**A**), rabbits treated with micafungin at 1 mg/kg as systemic therapy (MFG1) (**B**), lock therapy with micafungin at 10 mg/mL (Lock10) (**C**), or a combination of lock therapy with micafungin at 10 mg/mL and systemic therapy with micafungin at 1 mg/kg (Lock10+MFG1) (**D**). Values are means ± standard error of the means.

Untreated control rabbits exhibited a progressive increase in the fungal burden of blood from the CVC throughout the study, leading to disseminated disease in deep tissues (Figure 1A). Rabbits treated systemically with micafungin at a dose of 1 mg/kg (MFG1) demonstrated persistent presence of *C. albicans* in the catheter throughout the experiment (Figure 1B).

In contrast, rabbits treated with lock therapy alone using micafungin at 10 mg/mL (Lock10), or in combination with Lock10+MFG1, showed a rapid decrease in fungal burden from 5.0 × 10^4^ to 1.8 × 10^2^ CFU/mL (*p* ≤ 0.001) in the blood obtained from the CVC 24 h after the initiation of therapy (day 2 post-inoculation), achieving complete eradication of *C. albicans* 72 h after the initiation of therapy (day 4 post-inoculation) (Figure 1C,D).

#### 3.1.2. Efficacy of Lock Therapy and Systemic Therapy with Micafungin against *C. albicans*

**Figure 2:**
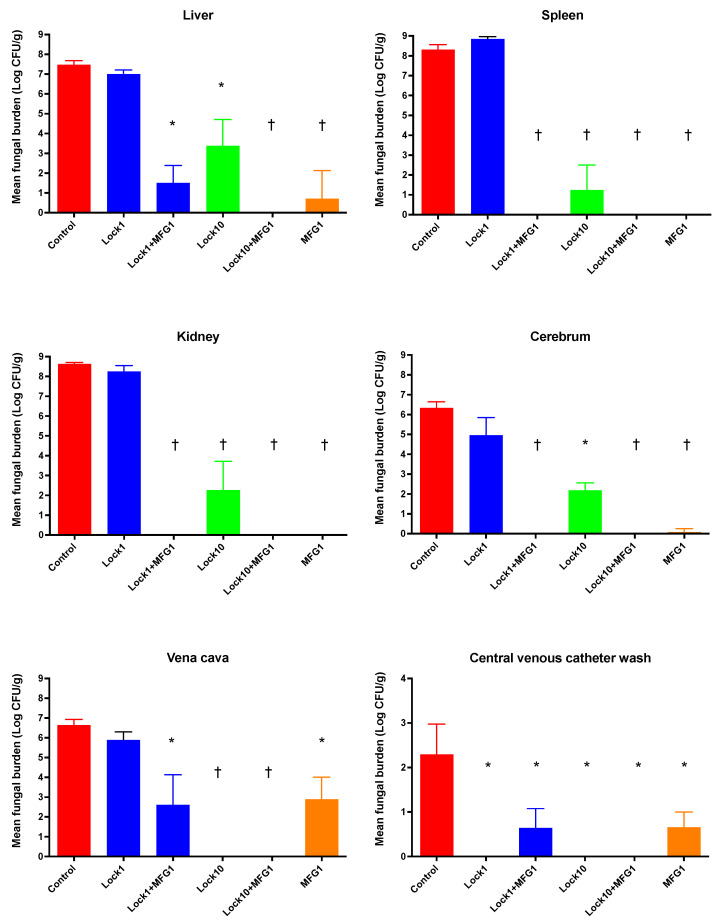
Responses of *C. albicans* fungal burden in disseminated candidiasis in persistently neutropenic rabbits to lock therapy and systemic antifungal therapy, measured by the determination of the mean log (CFU/g) concentrations of the organism in the liver, spleen, kidney, cerebrum, vena cava, and central venous catheter wash of untreated control rabbits and rabbits treated with micafungin as lock therapy at 1 mg/mL (Lock1), 10 mg/mL (Lock10), systemic therapy with micafungin at 1 mg/kg (MFG1), or the combination of Lock1+MFG1 and Lock10+MFG1. Values are means ± standard error of the means. * *p* < 0.05; † *p* < 0.01. *p*-Values are for the results of treated rabbits in comparison to untreated controls, as determined by ANOVA with Bonferroni’s correction for multiple comparisons.

Rabbits subjected to lock therapy with Lock10 and systemic therapy with MFG1 alone or in combination with Lock1+MFG1 displayed a significant reduction in fungal burden in all analyzed tissues, such as liver, spleen, kidney, cerebrum, and vena cava, and in the venous catheter wash (*p* ≤ 0.05) (Figure 2).

The combination of lock therapy and systemic micafungin therapy (Lock10+MFG1) achieved eradication of *C. albicans* from all studied tissues (0.0 ± 0.0 log CFU/g) vs. untreated controls (liver 7.5 ± 0.22, spleen 8.3 ± 0.25, kidney 8.6 ± 0.07, cerebrum 6.3 ± 0.31, vena cava 6.6 ± 0.29, and CVC wash 2.3 ± 0.68 log CFU/g (*p* ≤ 0.001).

#### 3.1.3. Assessment of (1→3)-β-D-Glucan Levels

**Figure 3:**
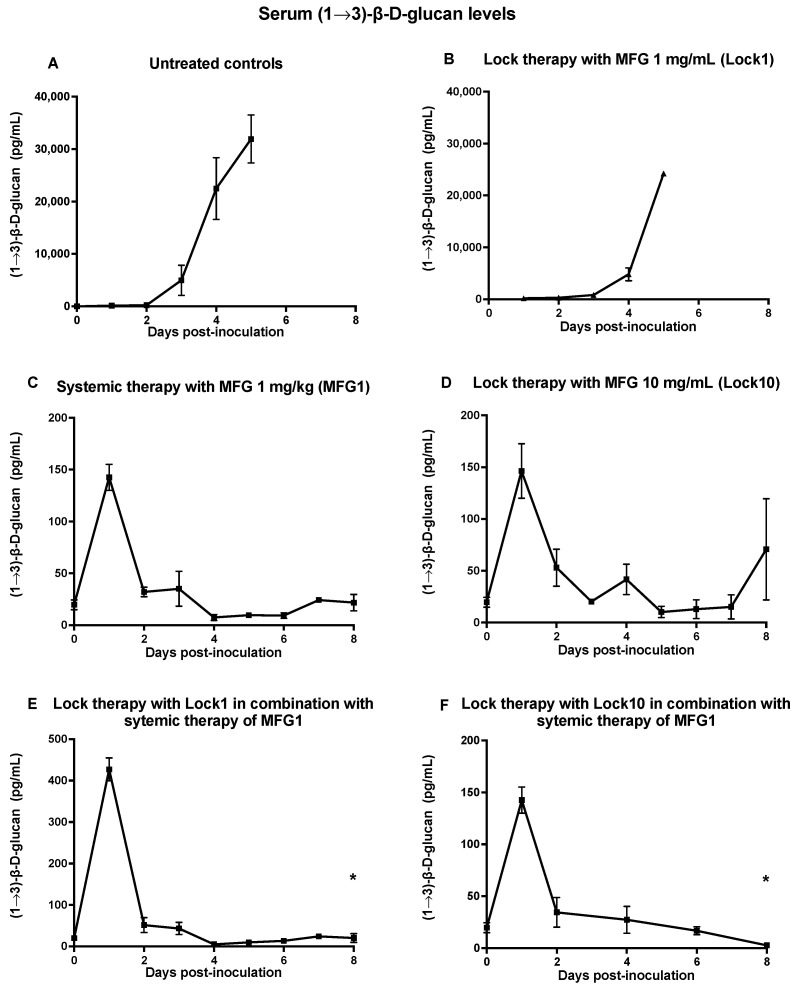
Expression of (1→3)-β-D-glucan levels in serum of persistently neutropenic rabbits with disseminated candidiasis caused by *C. albicans* in untreated control rabbits (**A**) and rabbits treated with lock therapy at 1 mg/mL (Lock1) (**B**) and 10 mg/mL (Lock10) of micafungin (**D**), systemic therapy with micafungin at 1 mg/kg (MFG1) (**C**), or a combination of Lock1+MFG1 (**E**) and Lock10+MFG1 (**F**). Values are means ± standard error of the means. * *p* < 0.05; *p*-values are for the results for treated rabbits in comparison to those for the untreated controls, as determined by ANOVA with Bonferroni’s correction for multiple comparisons.

Serum levels of (1→3)-β-D-glucan were monitored as a biomarker for the antifungal therapeutic response. Serum levels of (1→3)-β-D-glucan in rabbits treated with the combination of Lock1+MFG1 and Lock10+MFG1 showed a rapid and significant decrease by day 2 after inoculation, persisting in decline throughout the experiment (*p* = 0.05), and were consistent with the therapeutic response of micafungin in reducing the residual fungal burden of *C. albicans* in the tissues and serum (Figure 3E,F). Concurrently, serum (1→3)-β-D-glucan levels of untreated control rabbits and those treated with Lock1 demonstrated a consistent elevation (Figure 3A,B), whereas (1→3)-β-D-glucan levels of rabbits treated with a single lock therapy of Lock10 or systemic MFG1 were consistent with the combination therapy (Figure 3C,D).

### 3.2. Disseminated Candidiasis Caused by C. parapsilosis in a Neutropenic Rabbit Model

#### 3.2.1. Fungal Burden in Central Venous Catheter Blood

**Figure 4:**
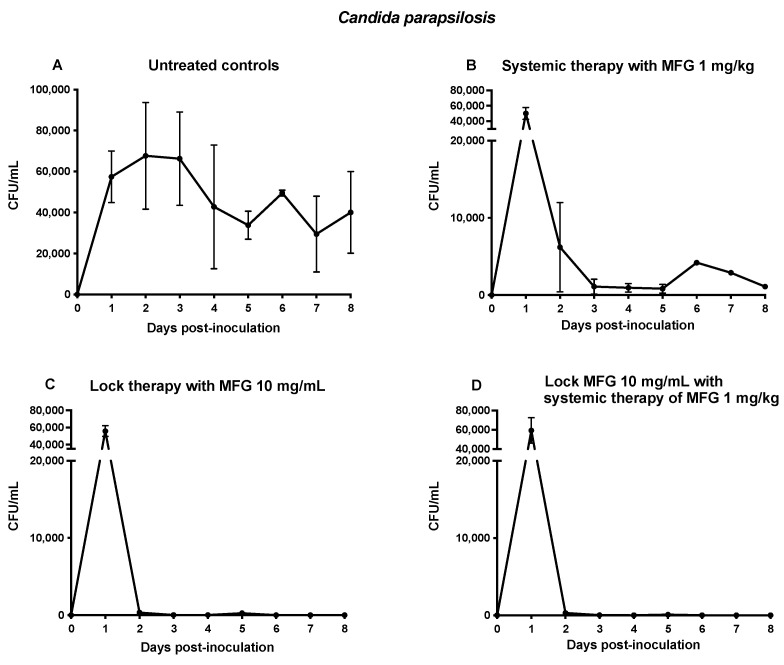
Responses of *C. parapsilosis* fungal burden in disseminated candidiasis in persistently neutropenic rabbits to lock therapy and systemic antifungal therapy measured by fungal burden in blood cultures from untreated control rabbits (**A**), rabbits treated with micafungin at 1 mg/kg as systemic therapy (MFG1) (**B**), lock therapy with micafungin at 10 mg/mL (Lock10) (**C**), or a combination of lock therapy and systemic therapy (Lock10+MFG1) (**D**). Values are means ± standard error of the means.

Blood cultures from untreated control rabbits in the persistently neutropenic *C. parapsilosis* disseminated candidiasis model demonstrated persistence of fungal burden in the CVC throughout the study (Figure 4A). Rabbits treated systemically with micafungin at a dose of 1 mg/kg demonstrated an initial increase in fungal burden, followed by a consistent decline during the experiment (Figure 4B).

In contrast, rabbits treated with Lock10 alone or in combination with Lock10+MFG1 demonstrated the most significant reduction in fungal burden from 5.9 × 10^4^ to 2.7 × 10^2^ CFU/mL (*p* ≤ 0.001) in the CVC 24 h after initiation of treatment and achieved complete clearance of *C. parapsilosis* 72 h after initiation of treatment (day 4 post-inoculation) (Figure 4C,D).

#### 3.2.2. Efficacy of Lock Therapy and Systemic Therapy with Micafungin against *C. parapsilosis*

**Figure 5:**
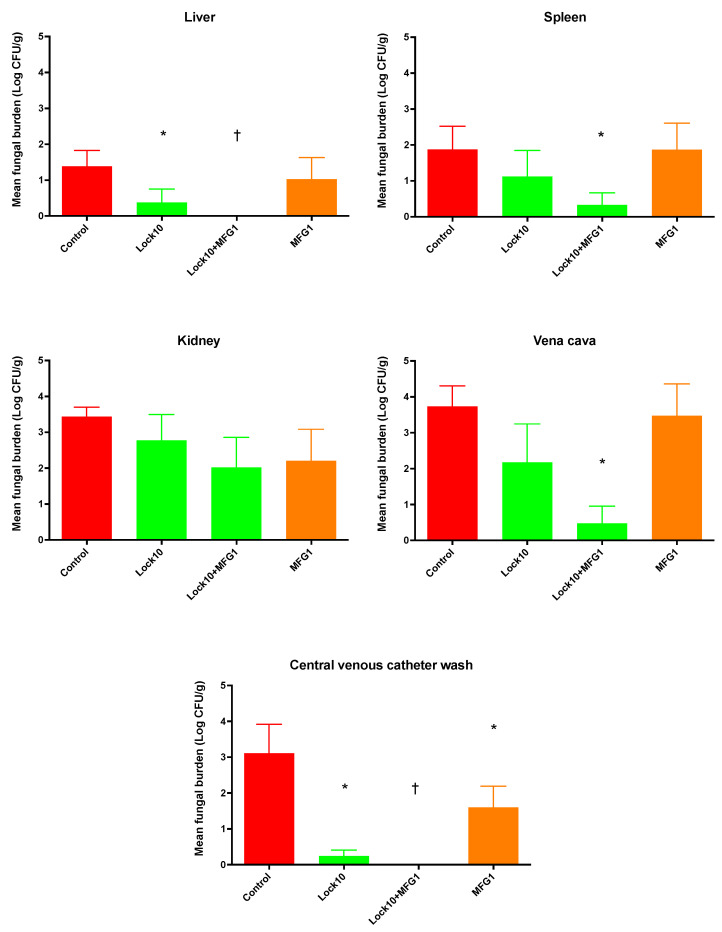
Responses of *C. parapsilosis* fungal burden in disseminated candidiasis in persistently neutropenic rabbits to lock therapy and systemic antifungal therapy, measured by determination of the mean log (CFU/g) concentrations of the organism in the liver, spleen, kidney, vena cava, central venous catheter wash, and vitreous humor of untreated control rabbits and rabbits treated with micafungin as lock therapy at 10 mg/mL (Lock10), systemic therapy with micafungin at 1 mg/kg (MFG1), or the combination of Lock10+MFG1. Values are as means ± standard error of the means. * *p* < 0.05; † *p* < 0.01. *p*-values are for the results for treated rabbits in comparison to those for the untreated controls, as determined by ANOVA with Bonferroni’s correction for multiple comparisons.

Rabbits treated with lock therapy Lock10 exhibited a significantly lower burden of *C. parapsilosis* in the liver and catheter wash (*p* ≤ 0.05) (Figure 5). Rabbits treated with a combination of lock therapy and systemic micafungin therapy (Lock10+MFG1) demonstrated a significant reduction in *C. parapsilosis* in the spleen and vena cava (*p* ≤ 0.05) but not in the kidney. Complete eradication of the organism during treatment with Lock10+MFG1 was achieved in the liver and CVC wash (*p* ≤ 0.01).

## 4. Discussion

This study demonstrated that lock therapy with micafungin eradicated *C. albicans* and *C. parapsilosis* from CVCs and that the addition of systemic micafungin significantly reduced the residual fungal burden in multiple deep tissues without CVC removal. Notably, lock therapy alone was not sufficient to eradicate deep tissue candidiasis, and systemic therapy with micafungin through the CVC did not eradicate *C. albicans* or *C. parapsilosis* from the catheter. These data provide preclinical evidence for the simultaneous administration of CVC lock therapy and systemic antifungal therapy with micafungin in profoundly immunocompromised animals for the treatment of *Candida* CRBSIs when the CVC is not able to be removed.

A discussion of the architecture and metabolism of *Candida* biofilms is helpful for understanding the tenacity of eradicating these structures within CVCs. Kuhn and colleagues characterized the biofilm formation of *C. albicans* and *C. parapsilosis* using a clinically applicable in vitro model of *Candida* biofilms on CVCs [12]. Using assays measuring tetrazolium (XTT) quantification, dry weight (DW), fluorescence microscopy, and confocal scanning laser microscopy with fluorescent probes, the authors found significant differences between invasive and noninvasive strains. They also observed that isolates of *C. albicans* produced more biofilm than those of *C. parapsilosis*, demonstrated by microscopy and DW. Biofilms produced by *C. albicans* displayed morphologic differences distinct from those of *C. parapsilosis* and other *Candida* species. The biofilm of *C. albicans* consisted of a basal layer of blastoconidia with an overlying dense matrix consisting of hyphae and exopolysaccharides. The FUN-1 fluorescent probe and XTT assay revealed that *Candida* biofilm cells were metabolically active. Cells comprising *Candida* biofilms developed fluconazole resistance (MIC > 128 μg/mL) rapidly within 6 h.

Nett and Andes reviewed the current understanding of the effect of the extracellular matrix of *Candida* biofilm on its pathogenesis [15]. The extracellular matrix material consists of a combination of exopolysaccharides, organism- and host-derived proteins, and lipids and DNA. The extracellular matrix of *Candida* biofilms facilitates evasion from innate host defenses and permits the emergence of antifungal resistance. The exopolysaccharide components of *Candida* biofilms are similar in composition to those of the *Candida* cell wall. For example, one large molecule consisting of approximately 12,000 residues containing (1,2) and (1,6) branched mannans are assembled with linear (1,6) glucans to form a mannan–glucan complex in the extracellular space. These exopolysaccharides serve as a sequestering barrier to the penetration of antifungal agents and promote the emergence of resistant cells. The protein component of *Candida* biofilms may serve to utilize the carbohydrates as an energy source or to propagate the dispersion of the extracellular matrix. The DNA component of *Candida* extracellular matrix biofilm is composed of non-coding regions and provides a molecular scaffold that also confers protection to the organism from antifungal agents and host defenses.

Micafungin and other currently licensed echinocandins (caspofungin, anidulafungin, and rezafungin) demonstrate potent in vitro and in vivo concentration-dependent antifungal activity, as well as a prolonged post-antifungal effect against medically important *Candida* spp. [32,38,39,40,41,42,43,44,45,46,47]. Ernst and colleagues demonstrated that echinocandins and amphotericin B exert a prolonged post-antifungal effect exceeding 12 h in vitro against *Candida* spp. when the concentrations exceed the MIC [38]. Ernst et al. also performed time-kill assays in RPMI 1640 medium with micafungin at 0.125 to 16 times the MIC(80) and demonstrated fungicidal activity at concentrations ranging from 4 to 16 times the MIC(80) against a range of *Candida* spp., including *C. albicans*, *C. tropicalis*, and *C. krusei* [40].

Micafungin, as well as the other echinocandins, also inhibit cell wall biosynthesis, the production of extracellular matrix, and the reduction in *Candida* biofilms [16,17,18,19]. These pharmacodynamic properties and anti-biofilm properties of micafungin provide a rational basis for its study in our rabbit model system against *Candida* CVC infection.

The intravascular exposure of *Candida* biofilm within the vascular catheter to extremely high concentrations (10 mg/mL) that are ≥10,000 times the MIC of micafungin against *C. albicans* and *C. parapsilosis* optimizes the C_max_/MIC ratio against these pathogens and inhibits biofilm formation. The results of the eradication of *Candida* from the catheter blood and the CVC postmortem wash validate this strategy of lock therapy.

In addition to maximizing the C_max_/MIC ratio to ≥10,000 times, there is also a time-dependent component to lock therapy. Our study demonstrates that transient exposure to an IV infusion of micafungin for the treatment of disseminated candidiasis is not effective in eradicating *Candida* from the CVC.

At the same time, as the elevated concentrations of micafungin within CVC lock therapy are not achieved in the plasma central compartment, there is no effect on the reduction in the residual tissue burden of *Candida* with this strategy alone. Simultaneous systemic therapy for treatment of disseminated tissue infection is required.

*Candida* CVC infection serves as a nidus from which hematogenous dissemination to deep tissues occurs. Systemic treatment with micafungin achieved significant reduction in deep tissues with the exception of the CVC. Only by combining lock therapy with systemic therapy of micafungin is control of CVC *Candida* infection and a reduction in deep tissue residual tissue candidiasis achieved.

There was expected variation within the inter-animal and inter-experimental variability of the CVC biofilm. Although a precise and consistent inoculum was administered to each animal, the variability of intralumenal CVC infection is reflected in the day 1 peak initial concentrations of *C. albicans* untreated controls in Figure 1 and throughout the course of monitoring of both *C. albicans* and *C. parapsilosis* untreated controls in Figure 1 and Figure 4, respectively. This variability in CVC *Candida* concentrations may be related to variations in the thickness and architecture of biofilm formation within the CVC. Despite this variability, the persistence of infection in untreated controls and the resolution of positive quantitative cultures in replicate experiments was consistently observed in the treated rabbits.

The CVC concentrations of *C. albicans* and of *C. parapsilosis* in the untreated rabbits varied over the course of time, perhaps reflecting the variable structural dimensions and development of *Candida* biofilm on the luminal surfaces. Despite the heavy burden of *Candida* within the CVC lumina, micafungin induced a rapid fungicidal reduction in viable organisms of >99.9% within the first 24 h and then complete eradication (>99.99%) within 48 h. These findings are consistent with in vitro time-kill assays of echinocandins exerting a rapid fungicidal effect on *Candida* spp. [29,38,39,40].

Rapid clearance of an infected CVC that is not removed is necessary in order to avoid sustained candidemia and continued hematogenous seeding of deep tissues. Systemic therapy alone through the CVC was less effective than lock therapy at rapidly clearing the CVC, especially in infection caused by *C. albicans*. Full CVC clearance was not achieved in *C. albicans* infection until day 6 and was never attained in *C. parapsilosis*. Thus, although the in vitro pharmacodynamics are clearly concentration dependent for micafungin, the CVC data indicate that there is also a time component required for the biofilm disruptive effects to occur.

Deep tissue sites, including liver, spleen, kidney, and cerebrum, were only successfully treated with full eradication of *C. albicans* to the lower limit of quantitation by systemic therapy. There was a dose–response relationship between the Lock1 and Lock10 groups, with greater deep tissue clearance occurring in the Lock10 study groups. This may be related to higher level of micafungin being released from the CVC lock therapy solution into the bloodstream’s central compartment.

The importance of combined lock therapy and systemic micafungin is illustrated in the deep tissue clearance of *C. parapsilosis*. The reduction in the residual fungal burden in hepatic, splenic, renal, and vena caval tissues was greater in the combination of lock therapy and systemic therapy. These data may reflect the role of CVC biofilm in propagating candidemia and sustaining deep tissue infection.

The clinical implications of these data are illustrated in several reported cases of treatment of *Candida* CRBSI. Two patients with *Candida* CRBSI or external ventricular drain infection have been managed successfully with simultaneous micafungin lock therapy and systemic antifungal therapy [48,49]. Piersigilli and colleagues from Bambino Gesù Children’s Hospital in Rome, Italy, reported the use of lock therapy with a solution of 0.3 mL of a 1:1 mixture of 70% ethanol and micafungin 5 µg/mL for the treatment of CRBSI caused by *C. albicans* in a male infant of 26 weeks’ gestation who required a chronic (360 day) indwelling CVC following complicated abdominal surgery [48]. The catheter was closed for 12 h, and systemic therapy with micafungin at 10 mg/kg/day IV was initiated in combination with liposomal amphotericin B at 5 mg/kg/day for 21 days. The fungemia was cleared and the CVC was saved.

Auriti et al., from the same neonatology team at Bambino Gesù Children’s Hospital, also applied the strategy of micafungin lock therapy to manage an infected external ventricular drain (EVD) by instilling micafungin at 1.5 µg/mL in sterile water, which was 100-fold higher than the MIC of the infecting isolate of *Candida albicans*; the EVD was then closed for 6 h with the micafungin solution [49]. Systemic therapy was administered as a micafungin 15 mg/kg IV loading dose followed by a 10 mg/kg/day IV maintenance dose plus flucytosine at 100 mg/kg Q12h orally. The EVD and cerebrospinal fluid were successfully cleared.

Caspofungin has also been used for simultaneous lock therapy and systemic antifungal therapy [50]. Ozdemir and co-authors described the successful treatment of a CRBSI caused by *Candida lipolytica* involving a Hickman catheter in a 9-year-old boy with relapsed neuroblastoma and an autologous stem cell transplant. Caspofungin was administered as simultaneous lock therapy and systemic antifungal therapy. The CRBSI was cleared and the Hickman catheter was saved. To our knowledge, there are no reported cases of anidulafungin administered to patients for simultaneous lock therapy and systemic antifungal therapy.

For the strategy of simultaneous lock therapy and systemic antifungal therapy with micafungin to be more widely adapted, prospective clinical trials are needed to establish this strategy for those unfortunate patients in whom CVC removal is not feasible for treatment of *Candida* CRBSIs.

## 5. Conclusions

This study investigated the effectiveness of combined systemic and lock therapies with micafungin for eradicating catheter-based biofilms and disseminated candidiasis caused by *C. albicans* or *C. parapsilosis* in persistently neutropenic rabbits. The combination of lock therapy and systemic micafungin demonstrated the most significant reduction in fungal burden in the CVC and deep tissues in parallel with significant declines in serum (1→3)-β-D-glucan levels.

## Data Availability

Data are contained within the article.
